# A Systematic Review of Neuroprotective Strategies in the Management of Hypoglycemia

**DOI:** 10.3390/ijms20030550

**Published:** 2019-01-28

**Authors:** Marius Nistor, Martin Schmidt, Isabel Graul, Florian Rakers, René Schiffner

**Affiliations:** 1 Department of Neurology, Jena University Hospital - Friedrich Schiller University, Jena 07747, Germany; marius.nistor@uni-jena.de (M.N.); florian.rakers@med.uni-jena.de (F.R.);; 2 Institute for Biochemistry II, Jena University Hospital - Friedrich Schiller University, Jena 07743, Germany; martin.schmidt@med.uni-jena.de; 3 Orthopedic Department, Jena University Hospital - Friedrich Schiller University, Campus Eisenberg, Klosterlausnitzer Straße 81, Eisenberg 07607, Germany; isabel.graul@med.uni-jena.de

**Keywords:** hypoglycemia, neuroprotection, diabetes

## Abstract

Severe hypogylcemia has been found to induce cerebral damage. While a number of illnesses can lead to hypoglycemic episodes, antidiabetic medications prescribed for glycemic control are a common cause. Considering the rising prevalence of diabetes mellitus in the population, we investigated neuroprotective strategies during hypoglycemia in the form of a systematic review in adherence to the PRISMA statement. A review protocol was registered in the PROSPERO database. A systematic literature search of PubMed, Web of Science, and CENTRAL was performed in September 2018. Based on a predefined inclusion protocol, results were screened and evaluated by two researchers. Both animal experiments and human studies were included, and their risk of bias was assessed with SYRCLE’s and the Cochrane risk of bias tools, respectively. Of a total of 16,230 results, 145 were assessed in full-text form: 27 articles adhered to the inclusion criteria and were qualitatively analyzed. The retrieved neuroprotective strategies could be categorized into three subsets: (1) Energy substitution, (2) hypoglycemia unawareness, and (3) other neuroprotective strategies. While on a study level, the individual results appeared promising, more research is required to investigate not only specific neuroprotective strategies against hypoglycemic cerebral damage, but also its underlying pathophysiological mechanisms.

## 1. Introduction

Despite decades of intense research and strict treatment regimes, diabetes mellitus (DM) remains an impactful diagnosis. Not only that, but the *Lancet’*s most recent “Global Burden of Disease Study” reported an increase from 2006 to 2016 both in the absolute number of DM-related deaths (31.1%) and the total years of life lost (25.3%) [1]. Furthermore, the International Diabetes Federation’s “Diabetes Atlas of 2015” estimated a rise in the global prevalence of DM from 8.8% in 2015 to 10.4% in 2040 [2]. The treatment and related costs of even the contemporary incidence rate of DM is estimated by Bommer et al. to amount to 1.8% of the global gross domestic product [3].

The potential complications of DM are manifold due to micro- and macrovascular pathophysiological processes that can lead to kidney disease, retinopathy, cardiovascular disease (CVD), and neuropathy, among others [4]. Due to the physiological and psychological consequences of the disease, DM patients oftentimes suffer from a lower quality of life (QOL) compared to the general population [5,6]. The desire to limit, and if possible avoid, the aforementioned complications of long-term DM oftentimes leads to strict glycemic control with glucose-lowering agents. This measure is accompanied by a risk of iatrogenic hypoglycemia, though, especially in intensively treated patients who receive insulin or sulfonylureas [7,8]. Varying estimates between studies attribute 4–10% of deaths in diabetic patients to severe (oftentimes nocturnal) iatrogenic hypoglycemic episodes [7]. Because of the subsequent development of decreased sympathoadrenal activity, even moderate recurrent episodes of hypoglycemia reduce the glucose threshold at which autonomic and neuroglycopenic symptoms are experienced by the patient and at which counter-regulatory physiological responses can be measured [8]. This state of impaired awareness of hypoglycemia (IAH) can, in combination with a defective glucose counter-regulation, lead to hypoglycemia-associated autonomic failure, a vicious cycle that exposes the patient to a 25-fold higher risk of further episodes of hypoglycemia [8,9].

Apart from the potential risk of death during severe hypoglycemic episodes, a further concern regarding recurrent episodes of hypoglycemia is cerebral damage. The results of many case studies and histopathological postmortem examinations have revealed that hypoglycemia can lead to cerebral decline, with the cerebral cortex, hippocampus, thalamus, and hypothalamus appearing to be the most vulnerable regions [10]. It has to be recognized, though, that these results originated from severe cases, sometimes in the context of protracted hypoglycemic comas that ultimately led to the death of the patients [10]. While these observations in the most severe cases might not allow easy conclusions about the influence of moderate or recurrent hypoglycemic episodes on cognitive decline and cerebral damage, a multitude of animal studies have revealed a connection between insulin-induced hypoglycemia and neuronal damage. This is potentially mediated through *N*-methyl-d-aspartate (NMDA)-receptor activation and oxidative damage, and is likely to be dependent on the extent (electroencephalographic isoelectricity being positively correlated to neuronal damage) and duration of the hypoglycemic episode [11]. Although not as clear as the results proposed by animal experiments, several studies have described a connection between hypoglycemia and cognitive decline in humans as well. Lee et al. reported a correlation between a history of severe hypoglycemic episodes and subsequent cognitive changes, a smaller total brain volume in magnet resonance imaging (MRI), and a prevalence for dementia incidence in a cohort study (a subset of the Atherosclerosis Risk in Communities (ARIC)) evaluating 2001 diabetic patients over a time span of 15 years [12]. Similarly, a Taiwanese seven-year follow-up study found a higher incidence rate of dementia in diabetic patients with a history of hypoglycemia [13]. In a 2015 meta-analysis of five studies, Mattishent and Loke furthermore presented evidence of a bidirectional interaction between the occurrence of hypoglycemic episodes and the incidence of cognitive impairment and dementia in older patients, with the former apparently increasing the risk of the development of cognitive decline and the latter predisposing patients to suffer from further hypoglycemic episodes [14]. 

Apart from an apparently deleterious influence on the central nervous system, hypoglycemic episodes furthermore possess the potential to damage the peripheral nervous system as well. The results of multiple animal studies have suggested that the peripheral nervous system is also susceptible to damage through hypoglycemia, such as axonal degeneration, demyelination, and microvascular changes [15]. To a certain extent, this damage has also been reported in humans suffering from long-term hypoglycemia and subsequently exhibiting distal sensorimotor neuropathy [10].

A further field of concern regarding potentially harmful hypoglycemic states is that of neonatal hypoglycemia, which can occur after birth due to a child’s conversion from an exogenous maternal glucose supply in utero to endogenous glycogenolysis and gluconeogenesis. Despite decades of research, though, there is no clear consent on treatment guidelines, and there is conflicting evidence on whether neonatal hypoglycemia actually correlates with cognitive or developmental deficiencies later in life [16].

In light of the rising incidence of diabetes and an aging population, research on and solutions for DM and its subsequent complications (as well as therapies for the side effects of the primary treatments) are becoming ever more important in order to avoid and lighten the burden experienced by patients as well as to mitigate the societal and economic strains caused by this illness. While one obvious solution for the potential cerebral dangers of hypoglycemia would be perfect glycemic control, Davis has noted that hypoglycemia most often occurs in intensively treated patients and is dependent on a multitude of medical and personal factors such as overinsulinization, missed meals, overstrenuous exercise, etc. [17]. An absolute avoidance of hypoglycemic episodes in antidiabetic therapy with today’s glucose-lowering agents therefore appears unrealistic due to the complexity of influencing factors. Additionally, the immediate therapy of hypoglycemic episodes—the administration of glucose—does not appear to prevent cerebral damage, as evidenced by the above-described studies. We therefore conducted a review of three medical databases in order to collect and summarize the current state of the research regarding potential neuroprotective strategies that might mitigate or avoid cerebral damage due to hypoglycemic episodes. A systematic literature search of PubMed, Web of Science, and CENTRAL was conducted in accordance with Preferred Reporting Items for Systematic Reviews and Meta-Analyses (PRISMA) guidelines in September 2018. As a result, we were able to retrieve 27 studies that met our inclusion criteria, which were qualitatively analyzed. To our knowledge, a review about this particular aspect of the side effects of hypoglycemia in the context of potential protective strategies has not been performed up to this point. Since we anticipated the number of results to be relatively low and we wanted to produce as comprehensive an overview of the current state of the research as possible, we decided to include both animal experiments and human studies. Our results yielded three subsets of neuroprotective approaches, namely strategies concerning energy substitution, methods to ameliorate IAH, and individual approaches.

## 2. Results

### 2.1. Study Selection

**Figure 1:**
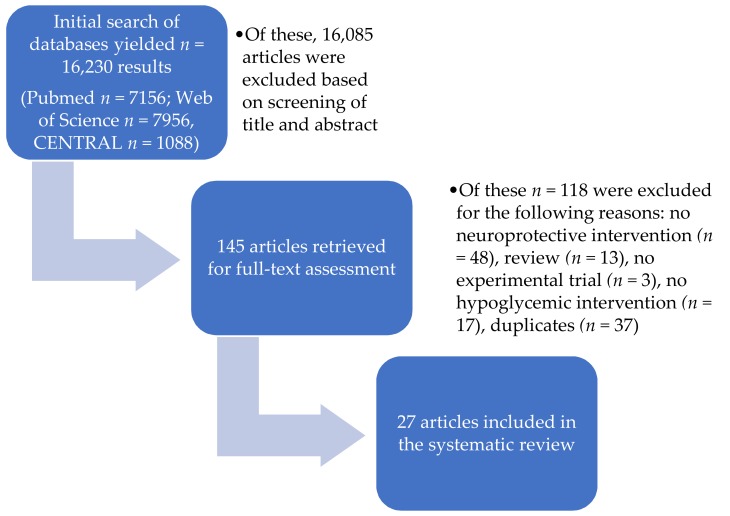
PRISMA flow chart.

In total, the comprehensive searches of the three databases, as described in detail in the “Methods” section, yielded 16,230 results. An initial screening of titles and abstracts based on our PICOS characteristics led to the exclusion of 16,085 studies. PICOS is a useful tool for asking focused clinical questions, e.g., P- Population or Problem, I- Intervention or Exposure, C- Comparison, O- Outcome, S- study design. The remaining 145 studies were retrieved and assessed in full-text form, which resulted in the subsequent exclusion of another 118 studies. The remaining 27 studies matched our inclusion criteria and were qualitatively assessed. The entire search and selection process is illustrated in the PRISMA flow chart (Figure 1).

### 2.2. Study Characteristics

All of the included studies were written in the English language and published at the time of this review. All of the human studies were performed in a clinical setting, and all animal studies were performed in a laboratory setting. 

Of the total of 27 included studies, 14 studies investigated conditions in human participants [18,19,20,21,22,23,24,25,26,27,28,29,30,31] and 13 trials were conducted on animals, of which 12 were performed on rats [32,33,34,35,36,37,38,39,40,41,42,43] and 1 on mice [44]. The average number of human participants was 15.3 ± 7.2, and the average number of animals utilized was 42.4 ± 17.8 (both as mean ± SD). All of the human participants were above 18 years old.

All studies utilized a predefined hypoglycemia model. The lowest blood glucose levels reached in human studies were within the range of 2.0–2.8 mmol/L, and eight studies utilized a stepwise reduction, therefore allowing a comparison of the effect of the intervention to varying degrees of blood glucose reduction [18,19,21,22,24,25,30,31]. In comparison to the human studies, the hypoglycemia models employed in the animal trials varied more significantly, with seven studies reporting defined blood glucose levels between <1–2.5 mmol/L [32,33,34,36,41,43,44], while the remaining studies defined the examined hypoglycemic episode as a specific time period of isoelectricity observed in electroencephalography (EEG) readings after insulin administration [35,37,38,39,40].

With regard to the neuroprotective interventions under investigation, the studies could be divided into three subsets: (1) Neuroprotection through energy substitution [18,19,20,32,33,34], (2) prevention through ameliorating the extent of impaired awareness of hypoglycemia [21,22,23,24,30], and (3) other neuroprotective interventions representing individual approaches without sufficient overlap to consolidate them into a more specific subset. The latter subset consisted of studies investigating hypothermia [35], adenosine triphosphate-sensitive channel potassium (KATP) modulators [25], target blood glucose level after a hypoglycemic episode [36], cyclosporine A [37,38], sleep deprivation [26], citociline [39], erythropoietin [27,28], alpha-amino-3-hydroxy-5-methyl-4-isoxazolepropionic acid (AMPA)- and NMDA-receptor antagonists [40], vitamin C and vitamin E [44], antecedent glycemic control [41], oral amino acids [29], chromium [42], memantine versus erythropoietin [43], and modafinil [31]. 

The parameters utilized to assess the influence of hypoglycemia on physiological processes and the effectiveness of the neuroprotective intervention varied widely. An overview of the general study characteristics is provided in Table 1, and an overview of the respective parameters used by individual studies is provided in Table 2.

### 2.3. Results of Individual Studies

#### 2.3.1. Approaches Regarding Energy Substitution

Chan et al. investigated the effects of local lactate delivery through microdialysis to the ventromedial hypothalamus (VMH) of rats in the context of counter-regulatory suppression of gamma-aminobutyric acid (GABA) and subsequent glucagon and epinephrine release. Preceding studies by the same research group had revealed that both recurrent hypoglycemia (RH) and diabetic rats showed increased extracellular lactate levels and higher extracellular GABA concentrations in the VMH. Chan et al. therefore hypothesized that in these conditions, the body’s adaption to, among other things, the alternate fuel substrate lactate and its subsequently elevated levels leads to counter-regulatory failure during RH. In acute hypoglycemic animals perfused with lactate, the administration of oxamate (OX, an inhibitor of lactate dehydrogenase) and alpha-cyano-4-hydroxycinnamate (4CIN, a lactate transport administrator) reduced VMH GABA concentrations and restored counter-regulatory responses compared to only lactate-infused animals, who showed a significant reduction of glucagon and epinephrine, whereas during the administration of the GABA_A_ receptor antagonist bicuculline methiodide (BIC), the animals exhibited normal counter-regulatory responses despite raised VMH GABA concentrations [32]. Animals with streptozotocin (STZ)-induced diabetes and preceding recurrent hypoglycemia regimens exhibited raised baseline GABA levels compared to controls, but also a restoration of counter-regulatory glucagon and epinephrine release after the administration of 4CIN and OX [32]. 

Choi et al. used an approach that included the consideration that not only the actual glucose decrease during a hypoglycemic episode is deleterious, but also the subsequent period after normalization of blood glucose levels. After hypoglycemia, due to glutamate and zinc release, elevated poly adenosine diphosphate (ADP) ribose polymerase (PARP) activation leads to energy depletion, lack of cytosolic Nicotinamid-Adenin-Dinukleotid (NAD), and therefore the inability to metabolize glucose even after it has been resupplied [33]. Since pyruvate metabolization does not depend on cytosolic NAD, Choi et al. administered pyruvate to diabetic rats after recurrent hypoglycemic episodes (one per day for five days). Compared to RH control rats, the animals treated with pyruvate exhibited less neuronal death as determined by Fluoro-Jade B (FJB) staining, less dendrite loss, and reduced zinc accumulation, oxidative injury, glutathione (GSH) loss, and microglial activation [33]. To mitigate pyruvate’s physiological relative inability to cross the blood–brain barrier, a plasma concentration of 5 mM was targeted, 100-fold higher than the physiological concentration [33]. 

Herzog et al. investigated the mechanism and extent to which adaption to lactate as an alternate fuel source after RH episodes might mitigate the effects of hypoglycemia and restore glucose metabolism to a certain extent. For that reason, rats that had undergone RH episodes in the previous three days (3dRH) were infused with [3-^13^C]-lactate during a hypoglycemic episode [34]. In contrast to control animals, 3dRH animals exhibited an elevated lactate uptake, glucose oxidization, and tricarboxylic acid (TCA) cycle. Since in preliminary experiments lactate was not able to contribute significantly to the TCA cycle during a resting state, the authors hypothesized that in the 3dRH animals, the modulated elevated lactate uptake during hypoglycemia provided extra oxidative energy for the neuronal TCA cycle with a subsequent increase in glucose uptake [34]. Rather than a primary alternative fuel source, lactate might therefore act as a metabolic modulator. 

Maran et al. investigated the effects of lactate infusion during hypoglycemia in seven healthy participants in a crossover trial three weeks apart, in which subjects received either lactate or saline 60 min before the first blood glucose reduction of a stepwise hypoglycemic clamp procedure [18]. Lactate reduced the glucose threshold at which four-choice reaction time deteriorated and delayed counter-regulatory hormone release of adrenaline, noradrenaline, growth hormone, and cortisol, while glucagon release remained unaffected. Furthermore, the emergence of autonomic and neuroglycopenic symptoms were delayed to lower blood glucose thresholds, as reported by the patients through a symptom questionnaire. An overview of the specific study characteristics and an overview of the respective parameters used by individual studies is provided in Table 3.

In a study based on their earlier research, Maran et al. conducted a crossover study in both healthy and type 1 diabetes mellitus (T1DM) volunteers. Both groups underwent a stepwise hypoglycemic clamp procedure with either administration of lactate or sodium chloride solution after the first observed neuroglycopenic symptoms, as reported by the patients [19]. No significant differences were observed between the time points of counter-regulatory hormone release, the beginning of neuroglycopenic symptoms, and the deterioration of four-choice-reaction time between the healthy and T1DM patients. Lactate infusion lowered epinephrine peak responses and blunted cortisol response, as well as ameliorated the severity of self-reported autonomic and neuroglycopenic symptoms in both healthy and T1DM participants [19]. Norepinephrine and glucagon release were unaffected by lactate infusion. In healthy participants, lactate prevented further deterioration of four-choice reaction time scores, while in T1DM participants, lactate led to complete recovery of cognitive function.

Page et al. investigated whether medium-chain fatty acids might mitigate hypoglycemic effects in a crossover study in which 11 T1DM participants received either medium-chain triglycerides or a placebo before a hypoglycemic clamp [20]. Utilizing a variety of cognitive tests (verbal memory, digit symbol coding, map searching, etc.), the authors reported a reversal of the impairment of cognitive performance observed during the placebo treatment after ingestion of triglycerides with higher levels of free fatty acids and ß-hydroxybutyrate. Catecholamines remained unchanged, however, as did the magnitude of self-reported autonomic and neuroglycopenic symptoms [20]. 

#### 2.3.2. Amelioration of Impaired Awareness of Hypoglycemia

De Galan et al. hypothesized that theophylline might be beneficial in diabetic patients with IAH, based on its blockade of central adenosine receptors with concomitant enhanced secretion of catecholamines and its reduction of cerebral blood flow (CBF) that might allow for an elevated sensitivity to decreasing glucose levels [21]. To verify this hypothesis, a crossover study was performed on 15 healthy participants and 15 T1DM participants with IAH, who on consecutive occasions received either intravenous theophylline or a placebo during a hypoglycemic clamp. Theophylline increased epinephrine, norepinephrine, and cortisol response in both participant groups and ameliorated hypoglycemia-induced CBF increase, albeit significantly more in T1DM participants. Furthermore, theophylline treatment shifted glycemic thresholds and the beginning of self-reported autonomic and neuroglycopenic symptoms to significantly higher blood glucose values in the T1DM group compared to the placebo, while no influence on these parameters was observed in the healthy participants [21]. 

Leelarathna et al. recruited participants for a 24-week trial targeting clinical strategies to identify blood glucose levels and avoid hypoglycemic episodes in the context of the restoration of hypoglycemia awareness [22]. For this purpose, 18 T1DM patients with IAH were assigned either into a group performing multiple daily injections (MDIs) with self-monitoring of blood glucose (SMBG), MDIs with SMBG and real-time continuous glucose monitoring (RT-CGM), continuous subcutaneous insulin infusion (CSII) with SMBG, or CSII with SMBG and RT-CGM [22]. Before and after the 24-week intervention period, hypoglycemic clamp procedures were performed. During the intervention period, incidences of biochemical hypoglycemia and blood glucose levels <3.9 mmol/l were reduced compared to the previous six months, as was the occurrence of severe hypoglycemia in both groups, without statistically significant differences between MDIs and CSII. In the post-intervention hypoglycemic clamp study, participants reported an earlier awareness of hypoglycemic symptoms compared to the baseline trial. Furthermore, metanephrine responses were elevated, while other counter-regulatory hormone levels remained unchanged, as did the threshold glucose level for cognitive deterioration [22]. While no clear statistical advantage of either MDIs or CSII (with or without RT-CGM) over each other could be observed, in general, efforts to provide a more specific glucose level adjustment with tighter controls were shown to have a mitigating effect on established IAH without loosening glycemic control. 

An overview of the specific study characteristics and an overview of the respective parameters used by individual studies is provided in Table 4.

In view of the relatively new and now widespread interest and participation in high-intensity interval training (HIIT), Rooijackers et al. investigated how elevated lactate levels after an HIIT session influenced cognitive, hormonal, autonomic, and neuroglycopenic responses to a subsequent hypoglycemic episode [23]. For this purpose, 10 healthy volunteers, 10 T1DM patients with IAH, and 10 T1DM patients with normal awareness of hypoglycemia (NAH) participated in a crossover trial on two occasions, undergoing a hypoglycemic clamp subsequent to either a bout of HIIT or a rest period. All patient groups exhibited a similar endogenous elevation of lactate levels after exercise, with a suppression of growth hormone and cortisol but unchanged catecholamine response as compared to rest. Only patients with T1DM and NAH exhibited statistically significant reduced neuroglycopenic and autonomic symptoms after HIIT as compared to rest, as well as ameliorated cognitive deterioration as determined by cognitive function tests. The authors hypothesized that in T1DM patients with IAH, no effect could be observed due to a “floor effect” of the established hypoglycemia unawareness that could not be surpassed.

Rossetti et al. compared human insulin to the long-acting insulin analogue detemir in 10 healthy participants in a crossover study in which each subject underwent an euglycemic clamp and a stepwise hypoglycemic clamp with either human insulin or detemir [30]. Detemir led to a greater response of autonomic and neuroglycopenic symptoms, albeit at lower blood glucose levels than human insulin, which would suggest a lowering of the awareness of hypoglycemia. Additionally, detemir exhibited greater cognitive deficits at higher blood glucose levels [30]. Counter-regulatory hormone levels did not differ between the two interventions.

With somewhat contrary results, Tschritter et al. equally compared the insulin analogue detemir to human insulin with regard to the sensitivity of hypoglycemic response and symptom awareness. Twelve healthy subjects underwent a stepwise hypoglycemic clamp in a crossover study with detemir or human insulin [24]. While no differences could be observed regarding counter-regulatory hormone response or cognitive function at respective glucose levels, symptom awareness as determined by a symptom questionnaire and sweating response exhibited elevated levels during detemir administration as compared to human insulin at the same blood glucose concentrations [24]. 

#### 2.3.3. Other Neuroprotective Interventions

##### Hypothermia

Agardh et al. examined potential neuroprotective effects of hypothermia [33] on hypoglycemia in rats, utilizing either halothane or isoflurane as anesthetic agents [35]. Morphological evaluation revealed a moderate region-specific protective effect on the caudoputamen and hippocampus, albeit only during halothane anesthesia. No neuroprotective effect was observed in isoflurane-anesthetized animals [35]. However, halothane animals did not regain spontaneous respiration and were euthanized after eight hours of artificial respiration, as compared to an observation period of one week in isoflurane animals [35].

##### Glibenclamide and Diazoxide

Bingham et al. investigated the influence of KATP-channel modulators on cognition and counter-regulatory hormone release during hypoglycemia in 10 healthy volunteers. Participants underwent a crossover trial of three hypoglycemic clamps, respectively with glibenclamide, diazoxide, or placebo administration, and one euglycemic clamp [25]. Autonomic and neuroglycopenic symptom intensity and thresholds as well as counter-regulatory hormone release did not differ between glibenclamide and diazoxide [25]. However, with glibenclamide treatment, preservation of cognitive function as assessed by four-choice reaction time was prolonged, with deterioration at 2.5 ± 0.3 mmol/L, as compared to diazoxide (3 ± 0.4 mmol/L) and a placebo (2.8 ± 0.5) [25]. 

##### Target Level of Blood Glucose Post-Hypoglycemia

Chu et al. investigated whether raising blood glucose levels above the physiological range post-hypoglycemia might lead to reperfusion injury in rats and to an exacerbation of neuronal damage [36]. Staining revealed moderate cell damage in hippocampal regions, with no significant differences regarding its extent in rats that were resupplied with glucose to target levels of either ≤3 mmol/L, ≤6 mmol/L, or ≤9 mmol/L [36]. In contrast, rats with a post-hypoglycemic blood glucose level of >9 mmol/L revealed significantly greater neuronal damage [36]. 

##### Mitochondrial Permeability Transition Mitigated by Cyclosporin A

Friberg et al. investigated the effects of cyclosporin A and FK 506 administration before a hypoglycemic clamp on sustained neuronal damages in rats [38]. Cyclosporin A, but not FK506, revealed a dose-dependent reduction of hippocampal brain damage, most likely through avoidance of mitochondrial permeability transition (MPT), since cyclosporin A-treated rats revealed reduced mitochondrial swelling as compared to FK 506 and control rats [38].

Based on the above-described results, the same research group expanded on their previous experimental model with a focus on apoptosis-inducing mechanisms subsequent to MPT. Control mice exhibited redistribution of cytochrome c at 30 min of EEG isoelectricity under hypoglycemia, and an increase in fodrin-breakdown products and active caspase-3 activity occurred 30 min to 3 h after normalization of blood glucose levels [37]. In rats that received prior administration of cyclosporin A, all of the above-described characteristics of cell decline were diminished in the hippocampal dentate gyrus region, less so in CA1 [37]. 

##### Influence of Sleep Deprivation on Hypoglycemia

Utilizing a number of cognitive tests, Inkster et al. investigated the effects of sleep deprivation on cognition and symptoms during a hypoglycemic clamp procedure. For this purpose, 14 T1DM patients underwent a hypoglycemic clamp in a crossover trial after either sleep deprivation for one night or a full night’s sleep [26]. While sleep deprivation did not significantly deteriorate the results of cognitive tests and symptom scores, sleep-deprived participants exhibited significantly poorer results in digit symbol substitution scores and choice-reaction times during the subsequent normoglycemic recovery period, as well as greater persistence of autonomic and neuroglycopenic symptoms [26].

##### Citociline

Kim et al. examined the effects of the acetylcholine precursor citicoline (cytidine 5′-diphosphocholine) on the extent of sustained neuronal damages in a rat model of 30 min of isoelectric hypoglycemia [39]. Compared to control animals, citicoline-treated rats exhibited reduced hippocampal neuronal death as quantified by FJB B staining, as well as ameliorated oxidative injury and microglial activation seven days after the hypoglycemic episode. Furthermore, the intervention revealed signs of blood–brain barrier stabilization, as citociline-treated rats exhibited less IgG leakage into the hippocampus than the control animals, as determined by immunohistochemistry [39].

##### Erythropoietin

Kristensen et al. investigated the physiological effects and correlation between renin-angiotensin system (RAS) activity, the endogenous release of erythropoietin (EPO), and cognitive function during hypoglycemia in a crossover study of induced hypoglycemia and maintained normoglycemia in nine T1DM patients with high RAS activity and nine T1DM patients with low RAS activity [27]. Normoglycemia revealed no significant increase in EPO in either patient group, while hypoglycemia triggered a significant rise in EPO in patients with high RAS activity, but no statistically significant rise in patients with low RAS activity. Lower EPO levels were correlated with poorer results in cognitive function testing during the hypoglycemic episode [27]. 

In a follow-up crossover study to evaluate the hormone’s potential neuroprotective effects, Kristensen et al. either administered exogenous EPO or placebo to 11 T1DM patients with IAH and recurrent severe hypoglycemia six days before a hypoglycemic clamp procedure design [28]. While EPO pre-treatment led to error reduction and less prolonged reaction time during hypoglycemia in reaction time tasks as compared to placebo treatment, no effect on other aspects of cognitive function (as assessed by the trail-making test and the Stroop color and word test), EEG recordings, or counter-regulatory hormones could be observed [28].

Silverstein et al. evaluated both the neuroprotective potential of memantine and EPO in a rat model undergoing a severe hypoglycemic clamp. Memantine-treated animals received an infusion immediately after 90 min of severe hypoglycemia, while EPO-treated animals received an intraperitoneal dose both 24 h before and after the experimental procedure, as well as an intravenous dose immediately after 90 min of severe hypoglycemia [43]. After an observation period of seven days, FJB staining revealed a cortical neuronal damage reduction of 35% in memantine-treated animals and of 39% in EPO-treated animals [43].

##### NMDA- and AMPA-Receptor Blockade

Based on previous research that showed that sustained post-hypoglycemic neuronal damage is at least partly caused by excitatory amino acids, Nellgård et al. investigated the effects of a blockade of the glutamatergic NMDA and AMPA receptors [40]. After a hypoglycemic clamp, rats received either the AMPA-receptor antagonist NBQX, the noncompetitive NMDA antagonist dizocilpine, a combination of the former, or the competitive NMDA-receptor blocker CGP 40116 [40]. Histopathological examination 3–4 days after the hypoglycemic clamp procedure showed a reduction of approximately 30% in striatal neuronal damages in all treatment groups as compared to control animals. Neocortical and hippocampal protection was achieved through combined NBQX and dizocilpine treatment, with a 50% reduction of neuronal loss, although results with single glutamate-receptor antagonists were varied [40].

##### Vitamins C and E Pre-Treatment

Patockova et al. investigated whether vitamins C and E, or a combination of both, could ameliorate oxidative stress as determined by malondialdehyde (MAD) after a hypoglycemic clamp procedure in mice [44]. Vitamins, both in monotherapy and combined, revealed statistically significant reductions in MAD concentrations post-hypoglycemia, suggesting a neuroprotective effect [44]. 

##### Antecedent Glycemic Control

Reno et al. investigated whether antecedent glycemic control prior to a hypoglycemic episode in STZ-induced diabetic rats ameliorated hypoglycemia-induced brain damage [41]. Diabetic rats were either left untreated or received daily insulin three weeks prior to the hypoglycemic clamp procedure. Subsequently, rats were either sacrificed after 1 week for brain histopathology, or performed cognitive testing (Morris water maze test) after 6–8 weeks [41]. Untreated diabetic rats displayed a 15-fold greater hippocampal and cortical extent of neuronal damage post-hypoglycemia as compared to nondiabetic hypoglycemic control animals, while in STZ-diabetic insulin-treated animals a significant reduction of neuronal damage was observed, nearly up to the level of control animals. Cognitive functional testing revealed no hypoglycemia-associated cognitive decline in any of the treatment groups [41].

##### Oral Amino Acid Administration in the Context of Glucagon Upregulation

Rossetti et al., based on the problem of loss of glucagon response in T1DM patients and preceding research that implied a stimulation of glucagon release after amino acid administration, examined whether an orally given amino acid mixture might positively influence glucagon release [29]. Utilizing a crossover study design, 10 healthy and 10 T1DM participants underwent two hypoglycemic clamps (amino acids and placebo) and one euglycemic clamp (amino acids) [29]. Oral amino acids administered during a hypoglycemic clamp raised glucagon levels in both healthy and T1DM participants. Although this increase was to a lesser extent in diabetic subjects, the glucagon levels observed nonetheless reached the concentrations exhibited by healthy participants to hypoglycemia during the placebo study [29]. Other counter-regulatory hormones were unaffected by the intervention in all groups, as were symptom score results. Cognitive test results exhibited partly elevated results after amino acid administration as compared to hypoglycemic placebo trials in both diabetic and nondiabetic subjects [29].

##### Influence of Chromium on Neuronal Plasticity Markers

Sahin et al. assessed the neuroprotective potential of chromium (Cr), in the form of Cr-histidinate (CrHis) and Cr-29 picolinate (CrPic) in a hypoglycemic rat model [42]. Both substrates, but especially CrHis, were correlated with enhanced expression of neuroplasticity markers [42]. 

##### Modafinil

Smith et al. investigated whether modafinil could enhance cognitive function and symptom awareness during a stepwise hypoglycemic clamp in a crossover study of nine healthy participants that either received modafinil or a placebo [31]. While counter-regulatory hormone responses were not altered by the drug, autonomic symptoms and heart rate were elevated in the modafinil-treated group. Furthermore, parts of the results of the cognitive function tests revealed an amelioration of hypoglycemia-induced cognitive deficits in the modafinil-treated group [31].

An overview of the specific study characteristics and an overview of the respective parameters used by other neuroprotective interventions is provided in Table 5.

### 2.4. Risk of Bias within Studies

The risk of bias of the retrieved animal studies was assessed with Systematic Review Centre for Laboratory animal Experimentation (SYRCLE’s) risk of bias tool [45]. The manner of the assessment process is described in the “Methods” section. 

Regarding the individual categories of interest of SYRCLE’s risk of bias tool, none of the included animal studies showed a low risk of bias regarding sequence generation, all of the studies reported baseline characteristics, none of the studies showed a low risk regarding allocation concealment, none of the studies reported efforts regarding random housing (this category was not applicable to one study), 30% of the studies showed a low risk of bias regarding blinding to the intervention, 61% of the studies showed a low risk regarding random outcome assessment, 69% of the studies showed a low risk of bias regarding blinding to outcome, all studies showed a low risk of bias regarding both incomplete outcome data and selective outcome reporting, and 69% of the studies showed a low risk regarding other sources of bias. 

Overall, risk of bias across studies was medium to low, albeit a number of studies exhibited an unclear risk of bias in several categories due to insufficient reporting. Five studies mentioned the occurrence of randomization [36,41,42,43,44], but none adequately specified the specific procedure that was used (sequence generation) nor whether concealment measures were taken (allocation concealment). Similarly, while the majority of studies provided details about animal housing [32,33,34,37,38,39,40,43], no information was provided whether the personnel were blinded to treatment groups or randomization efforts were made in the keeping of the animals. Nonetheless, all studies reported baseline characteristics and had a low risk of bias regarding incomplete outcome data and selective outcome reporting. In general, we found no gross systematic faults or discrepancies that would definitely point toward a high risk of bias in the majority of studies, rendering overall risk of bias across studies as medium (or rather unclear, due to the lack of information especially regarding randomization and blinding). 

All individual results of SYRCLE’s risk of bias tool are visualized in Table 6.

To assess the risk of bias of the human studies, we utilized the Cochrane risk of bias tool [46].

Focusing on the individual categories of the risk of bias tool, 40% of the studies exhibited a low risk of bias regarding random sequence generation, 13% exhibited a low risk regarding allocation concealment, 80% showed a low risk of bias regarding blinding of participants and personnel, all studies showed a low risk of bias regarding blinding of outcome assessment, 86% showed a low risk of bias regarding incomplete outcome data, 93% showed a low risk of bias regarding selective reporting, and 86% exhibited low risk regarding other biases. 

Overall risk of bias across studies was medium to low. The studies of Kristensen et al. [28], Maran et al. [19], and both studies by Rossetti et al. [29,30] exhibited generally low risk of bias, and the rest of the studies showed a medium risk of bias. While all studies mentioned randomization procedures, only the aforementioned specified these with additional information regarding the actual randomization process. Overall, none of the included studies exhibited an explicitly high risk of bias, although most of the studies exhibited a medium risk of bias, though mostly based on lack of provided information and not due to obvious systematic faults. 

All individual results of the Cochrane risk of bias tool are visualized in Table 7. 

## 3. Discussion

A systematic literature search of three medical databases for studies concerning neuroprotective interventions during hypoglycemic states yielded a total of 27 results, 14 of which were human studies and 13 of which were experimental trials in animals. 

### 3.1. Retrieved Studies

Alternative energy sources during states of hypoglycemia have been discussed and investigated for a number of years with differing results. Four of the studies we identified investigated the influence and potential of lactate during hypoglycemic states on the brain. For the longest time, lactate was only considered a marker of cell hypoxia [47]. In recent decades, however, lactate has been proposed to have beneficial effects in a number of neurological diseases. While the exact mechanisms are still controversially discussed, the astrocyte neuron lactate shuttle theory has been proposed as a possible means of the brain to utilize lactate with a subsequent glucose-sparing effect [48]. Lactate furthermore has been associated with signaling functions on vasodilation (increased CBF) and axonal regeneration [47]. As a consequence, lactate is under investigation for its potential neuroprotective effects in stroke [48], traumatic brain injury [49,50], retinal neurodegeneration [51], and hypoglycemic states of differing origins. Four of the retrieved studies in this review examined the effects of lactate during hypoglycemic episodes. Herzog et al.’s results in rats certainly pointed toward a potential intrinsic neuroprotective adaptive process through previous hypoglycemic episodes, which subsequently leads to an increased lactate uptake [34]. Nonetheless, Maran et al. observed a beneficial effect on cognitive function and a lowering of the glucose threshold at which autonomic and neuroglycopenic symptoms were experienced in humans both when lactate was administered before the hypoglycemic clamp procedure [18] and also when it was only infused after the first emergence of hypoglycemic symptoms [19]. The delay of counter-regulatory hormones that was observed in both of Maran et al.’s studies after lactate infusion was further addressed in Chan et al.’s study. According to Chan et al., counter-regulatory failure and recurrent hypoglycemic episodes could be due to an elevated GABA release caused by increased lactate levels that provide substrates for GABA synthesis and also, through the provision of energy to neurons, mask the body’s glucose deprivation [32]. The latter might be indicative that VMH GABA-ergic neurons may not be as glucose-sensitive as suspected, but rather serve as general metabolic fuel sensors [32]. The (adaptive) utilization of lactate as an alternate metabolic fuel in diabetic patients has been proposed as one potential mechanism for the development of impaired awareness of hypoglycemia (IAH) and as a contributing factor to hypoglycemia-associated autonomic failure (HAAF) [52,53]. While lactate might provide neuroprotective effects, either through metabolic modulation or as a primary alternative metabolic fuel, further research is needed to investigate its exact mechanisms. Furthermore, lactate’s connection to the occurrence and development of IAH should be a focus of future research, since potential therapeutic applications would have to be weighed against such side effects. 

Page et al. and Choi et al. investigated additional approaches regarding energy substitution, through medium-chain fatty acids and pyruvate administration, respectively [20,33]. Through metabolization into ketones and dose-dependent uptake through the blood–brain barrier, exogenously supplied lipids might be able to support the brain during energy depletion and provide a glucose-sparing effect. Further research into the extent of this protection is necessary, though, as are investigations into potential pitfalls, such as a certain necessary glucose level to provide the substrates of the TCA cycle to further metabolize ketones into acetyl-COA in the brain, as noted by Amaral [54]. Pyruvate might be beneficial as an alternative energy substrate since it is still able to be metabolized even after post-hypoglycemic PARP-1-induced cytosolic NAD depletion that inhibits glucose utilization [33,55]. In the context of its glucose-sparing effect, pyruvate has additionally been investigated in such conditions as forebrain ischemia [56] and hemorrhagic shock [57]. While potentially promising, further research is needed. Indeed, Choi et al. noted that their utilized dosage represented the 100-fold physiological level of pyruvate [33]. As Amaral observed, a number of studies have not been able to achieve a significant elevation of measured brain pyruvate levels through oral administration, and a number of potential side effects, such as lactic acidosis (through conversion into excess lactate) or metabolic alkalosis by large doses of pyruvate, need to be further investigated [54].

Depending on the magnitude of responsiveness, disease progression, and the need for exogenous glycemic control, the subsequent choice of which therapeutic antidiabetic drugs to prescribe a patient also carries differently weighted risks concerning the occurrence of hypoglycemic episodes. Insulin and sulfonylureas are considered to be the most prone to exhibit these side effects [58]. The question of which form of insulin is superior regarding both glycemic control and reduction of hypoglycemic risk is an ongoing topic. Tschritter et al. and Rossetti et al. have both examined the long-acting basal insulin analogue detemir in comparison to human insulin with somewhat differing results: Tschritter et al.’s results implied a positive effect on hypoglycemia awareness, and Rossetti et al.’s implied the reverse [24,30]. Tschritter et al. argued that this discrepancy was due to differences in experimental design regarding glucose infusion rates and insulin regimens during the insulin-induced hypoglycemic clamp [24]. In a comprehensive review published in 2011, Little et al. evaluated a number of studies comparing NPH insulin and insulin detemir regarding the risk of hypoglycemia, with detemir administration exhibiting an ameliorating effect on the number of hypoglycemic episodes in the majority of studies in both T1DM and T2DM patients [59]. Nonetheless, Little et al. noted that in the majority of studies, patients with a history of IAH or severe hypoglycemic episodes were excluded [59]. A study by Strandberg analyzing a Swedish population of 16,985 persons, which similarly showed a significantly lower risk of severe hypoglycemic episodes under detemir treatment as compared to human insulin (and glargine), equally only regarded patients that were recently prescribed insulin [60]. Therefore, more research is needed specifically targeting the most vulnerable patient group, which suffers from long-duration diabetes with recurring hypoglycemic episodes and established IAH. 

Since repetitive hypoglycemic episodes represent one mechanism for, and elevate the risk of, development of IAH, strict glycemic control has to be balanced between achieving stable blood glucose levels without increasing the risk of hypoglycemia [61]. Continuous subcutaneous insulin infusion and continuous glucose monitoring have both been found to improve glycemic control without worsening hypoglycemia occurrence: Indeed, CSII has been associated with lower rates of (nocturnal) hypoglycemia [61,62,63]. While Leelarathna et al. did not observe a significant difference between multiple daily injections (MDIs) of insulin and CSII (with or without real-time continuous glucose monitoring), tightening of the observation of blood glucose levels achieved a reduction of hypoglycemia incidence without a worsening of glycemic control [22]. The reported positive influence on hypoglycemia awareness in the post-intervention hypoglycemic clamp procedure underscored not only the significance of avoidance of hypoglycemia as a preventive measure against the development of IAH, but also the significance of it as a corrective measure once IAH has been established [22]. 

Rooijackers et al.’s experimental study evaluated the effects of high-intensity interval training (HIIT) on subsequently reduced reactions to hypoglycemia through lactate elevation. While exercise has been shown to induce altered counter-regulatory responses to subsequent hypoglycemia, the magnitude of this effect seems to be dependent on the intensity and duration of the exercise [64,65,66]. Rooijackers et al.’s results pointing toward the vulnerability of T1DM patients with normal awareness of hypoglycemia to high-intensity exercise therefore reveals the need for further investigation into the effects specific exercise regimens have on diabetic patients [23]. Additional results would allow relevant advice to be given to specific patient groups concerning the sort and extent of exercise that can be recommended, and which should be avoided.

The neuroprotective properties of erythropoietin have been investigated for a number of years, regarding, for example, stroke and Alzheimer’s disease [67], autoimmune encephalomyelitis [68], neuroprotection in pre-term infants [69], as well as other neurodegenerative and traumatic diseases with cerebral damage such as Parkinson’s disease, traumatic brain injury, and cardiac arrest [70,71]. Kristensen et al.’s two studies observed both an endogenous (correlated to baseline RAS activity) and exogenous neuroprotective effect on cognitive function (endogenous) and reaction time (exogenous) [27,28]. Silverstein et al.’s rat model revealed a reduction in neuronal cell damage as determined by FJB staining [43]. While a number of animal studies have yielded positive results, a multicenter study did not observe any neuroprotective results in early erythropoietin administration after out-of-hospital cardiac arrest, but rather a higher number of serious adverse events in the erythropoietin-treated group, especially of a thromboembolic nature [71]. More research is warranted regarding effective dosage, treatment regimens, and the newly derived isoforms of erythropoietin that could exert neuroprotective properties without hematopoietic effects [67,70]. 

While Agardh et al.’s hypothermic intervention showed some neuroprotective effects, it seemed to be rather correlated to the utilized anesthetic agent (which in turn was correlated with an inability to resume spontaneous respiration) [35]. Furthermore, while hypothermia has yielded positive neuroprotective results in a variety of animal disease models, these results have not been successfully reproduced in humans [72]. 

The advantage of strict glycemic control is mostly defined as the avoidance of diabetic complications such as micro- and macrovascular complications, cardiovascular disease, etc. [4]. The results by Reno et al. point toward an additional utility of glycemic control, as in the rat model used, untreated diabetic rats exhibited greater neuronal damage after a hypoglycemic episode than insulin-treated diabetic rats [41]. The potential importance of defining specific beneficial (normoglycemic) blood glucose levels on a daily basis, but also during specific situations such as reperfusion after a hypoglycemic event, was emphasized by the results of Chu et al. [36]. Glucose reperfusion post-hypoglycemia has previously been shown to be potentially detrimental due to extracellular zinc release and the activity of NADPH oxidase [73]. Chu et al.’s results similarly indicated that post-hypoglycemic reperfusion of neuronal cells above blood glucose levels of 9 mmol/L might exert damage through such mechanisms [36]. 

The experiment of Inkster et al. concerning the effects of sleep deprivation on symptoms and cognitive functions during hypoglycemia was certainly interesting, since knowledge of a potential deleterious effect might serve to mitigate the danger of hypoglycemic episodes by prevention, since clear advice regarding the adaption of an adequate sleep pattern could be provided to the patient. While Inkster et al. only observed a deleterious effect of sleep deprivation during the normoglycemic recovery period after the hypoglycemic clamp procedure, the authors themselves mentioned that since they did not monitor counter-regulatory hormone levels, their study cannot be seen as providing a complete answer regarding the research question [26]. Furthermore, the study utilized a mixed participant group of night-shift workers and non-night shift workers, so that some of the participating individuals might have developed circadian adaptions [26].

A number of studies we were able to retrieve used drugs or mechanisms aiming at different targets in the metabolism of blood glucose and cell metabolism in general, as well as its resulting pathophysiological alterations either after long-term illness or acute hypoglycemic depletion. Cyclosporin A, via avoidance of mitochondrial permeability transition (MPT), revealed promising results in rats by ameliorating observed mitochondrial swelling and subsequent signs of cell decline [37,38]. Cyclosporin A has furthermore been shown to exert neuroprotective effects through avoidance of MPT in both adult and immature animal models of traumatic brain injury [74]. Bingham et al. hypothesized that glucose-sensing neurons of the hypothalamus might share mechanisms with pancreatic ß-cells and that glibenclamide might be able to exert an effect in those regions as well [25] While Bingham et al.’s study was not able to elucidate the exact mechanisms behind this observation, a neuroprotective or sparing mechanism could be conjectured, since a delayed deterioration of choice reaction time was observed [25]. This potential positive effect, however, was marred by research that showed that newer-generation sulfonylureas possessed a lower risk of hypoglycemia and that glibenclamide inhibited glucagon counter-regulation [58,75]. Further approaches that we were able to identify through our systematic literature search were NMDA/AMPA blockades [40], glucagon upregulation by amino acid administration [29], citociline [39], chromium [42], vitamins C and E [44], and modafinil administration [31]. While individually promising, further research into the potential neuroprotective properties of these substances is needed, as well as their potential side effects. Although well researched, not all mechanisms that influence and propagate cellular damage through hypoglycemia are currently known, and neither are the regulatory and adaptive processes of glucose regulation [76]. Additional research into these physiological and pathophysiological processes will ultimately facilitate the direction toward which future neuroprotective strategies will develop.

### 3.2. Study Quality and Translational Value

The majority of both the human studies and animal experimental studies exhibited a medium risk of bias. In both human and animal studies, the reporting of appropriate measures against selection bias was very imprecise and therefore prevented an accurate evaluation of this important aspect. Only five of the animal studies reported randomization, but none of them specified the randomization procedure, and while all of the human studies reported randomization, only a minority specified the exact steps taken to avoid selection bias. Kahan et al. performed a screening of 152 trials, the majority of which exhibited inadequate reporting or utilized measures to limit selection bias such as appropriate randomization and allocation procedures and blinding of participating personnel [77]. The reviewed studies within this review exhibited a similar pattern: Avoidance of selection bias and especially detailed reporting of the utilized measures should be employed in the future to ensure that readers are able to evaluate study quality.

The majority of the included animal studies utilized extended observation periods, but none reported in a detailed way whether animals were randomly housed or whether the veterinary personnel were blinded to the received intervention during the subsequent observation period. Since potential unconscious differences in the treatment of the animals or even positions within the facility (temperature, light, etc.) might exert an influence on the observed results [45], efforts should be made to randomly house animals during the trial and subsequent observation period, and this should be reported in detail.

Heller and McDonald wrote as far back as 1996 that the assessment of cognitive function during hypoglycemic trials and the translational value of studies is potentially limited due to the multitude of different cognitive tests and symptom questionnaires that are employed to assess deteriorating cognition in human studies [78]. While there was an overlap regarding the utilized cognitive function tests between some of the human experimental studies, future experiments would benefit from a set of standardized tests and experimental protocols regarding the assessment of cognitive function (as well as similar hypoglycemic clamp procedures) to strengthen interstudy validity and translational significance.

### 3.3. Limitations

The aim of this systematic review was to provide a wide overview into current approaches and the current state of research of neuroprotective strategies and interventions during hypoglycemic episodes. Our systematic search was therefore designed to be broad and to include as many related articles as possible. Consequently, we retrieved a variety of differing approaches and were able to qualitatively assess them. Nonetheless, this very inclusive approach might have also led to the preclusion of some results that might have been yielded if a search protocol were used that was more specifically focused on some of the subsets we identified, for example energy substitution or hypoglycemia unawareness. While we are confident in presenting a somewhat comprehensive summary of the review question, we cannot preclude the possibility that due to the broadness of our search protocol, some eligible studies were not covered. Furthermore, we were not able to perform an EMBASE search, since we were not able to gain access to this database through the means supplied by our institution.

Due to the broadness of the research question and the subsequently wide-ranging and differing approaches (regarding the sorts of interventions, methodologies, and the investigated subjects, i.e., humans or animals), we did not perform a quantitative analysis. While some subsets within the retrieved studies could be identified, due to the aforementioned differences this review format did not lend itself to a statistical analysis. Future reviews might quantitatively evaluate the herein identified subsets through a more specified search protocol. As a consequence of this methodological choice, this review should not be understood as providing treatment advice or recommendations, but rather as providing a general overview regarding the current state of research. 

## 4. Materials and Methods

### 4.1. Review Protocol

As an initial step of the work process of this systematic review, we prepared a review protocol that already outlined all PICOS characteristics, included a provisory search strategy, and specified the data collection and data synthesis process, as recommended by the PRISMA-P statement [79,80]. The review protocol was published in the University of York’s PROSPERO database (ID: CRD42018115479).

### 4.2. Eligibility Criteria

The eligibility criteria were as follows:

1. Participants: For humans, participants with hypoglycemia/hypoglycemic episodes that were either not related to an established illness or related to a T1DM or T2DM diagnosis. Both sexes over the age of 18 years were included. For animals, animal experimental models that investigated isolated hypoglycemic episodes and hypoglycemia due to T1DM and T2DM, respectively, and antidiabetic medication, with the inclusion of all species and both sexes;
2. Intervention: All neuroprotective interventions;
3. Comparators: Usual care/normal practice, no intervention/control group, other neuroprotective interventions (if the respective study contained multiple interventions);
4. Outcome measures: Neuroprotective effects;
5. Study design: Controlled trials.

Basic limitations placed on the considered studies were time (published between 1990 and 2018 up to the performance of the systematic search in September 2018) and language (English). 

No specific limits were defined for the duration and depth of the hypoglycemia intervention. As our definition of the comparators specified at least one other experimental group, we judged that a reported neuroprotective effect in one experimental group as compared to the other ensured that the respective study’s experimental design employed a sufficient hypoglycemic depth to induce either cerebral damage (animal experiment) or a sufficient trend of declining scores in cognitive tests or investigated parameters (human studies) against which the intervention could be validated. Similarly, in order to be as inclusive as possible regarding the employed study design, we decided against defining a temporal limitation of the intervention’s administration with respect to the hypoglycemia experiment.

Primary exclusion criteria were aspects in the investigated studies that did not adhere to our PICOS criteria. With respect to the category of “participants”, we formulated further specific exclusion criteria in order to ensure that the investigated neuroprotective strategy and its analyzed effect would not be confounded by coexisting conditions that additionally influenced or altered cerebral physiology. Studies that observed human participants with a history of other illnesses, as reported by the authors, that potentially alter cerebral physiology, such as stroke, traumatic brain injury, epilepsy, neurodegenerative diseases, etc., were therefore excluded. Similarly, animal studies that utilized experimental models that, besides hypoglycemia, featured further illness models with a potential pathological influence on cerebral physiology (ICB, stroke, TBI, etc.), or that examined neuroprotective interventions in hypoglycemic episodes that were caused by a primary illness with an influence on cerebral physiology itself, were excluded.

### 4.3. Information Sources and Systematic Search

A comprehensive literature search of the PubMed, Web of Science, and CENTRAL databases was performed in August 2018. Pre-defined search strategies were utilized that were adapted to the respective database’s search algorithm. Where appropriate and possible, MeSH terms were used. Search terms included word and thematic variations of the terms “hypoglycemia”, “neuroprotection”, and “controlled trials”. Language (English) and time of publication (1990–2018) filters were employed to limit the results to the basic inclusion criteria. The complete search protocols can be accessed in Appendix A.

### 4.4. Study Selection

The complete results yielded by the systematic search of all three databases were examined for eligibility in this review in an unblinded manner by two researchers. The titles and abstracts of studies were initially screened. Thereafter, the remaining studies’ full-text manuscripts were accessed and evaluated based on the above-defined inclusion and exclusion criteria. Disagreements in the study selection process were resolved through discussion and consensus.

### 4.5. Data Collection Process

In order to uniformly extract the desired data from the retrieved studies, a data extraction sheet, based on the “Joanna Briggs Data Extraction Form for Experimental and Observational Data” and adapted for our individual purposes, was created [81]. Data were extracted by two researchers in an unblinded manner. Disagreements were resolved through discussion and consensus. We described previously [82].

### 4.6. Data Items

The following data were extracted from the included studies:

1. Basic information about the study (author, year of publication);
2. General characteristics of the experiment (number of participants/animals, setting) and its participants, including age (mean/SD), gender, and medical history as reported by the authors in human studies and species, gender, age, information regarding housing and keeping, and illness model in animal studies;
3. Information regarding the hypoglycemic intervention, including the manner of induction and the depth and duration of hypoglycemia;
4. Information regarding the neuroprotective intervention, including the time and duration/number of applications and the dosage;
5. Information regarding the employed outcome parameters by the respective study, including all relevant vital parameters, blood values, cerebral and cognitive outcome measures (histopathological, imaging, brain-specific parameters, cognitive tests, etc.), and the length of the observation period subsequent to the intervention.

### 4.7. Risk of Bias in Individual Studies

Since this review included both human studies and animal experimental studies, two different measures to evaluate the risk of bias of the respective studies were utilized. Human studies were evaluated with the Cochrane risk of bias tool [46] and animal experimental studies with SYRCLE’s risk of bias tool [45], the latter being based on the former but specifically refined for animal studies to include categories such as animal housing. All studies were assessed independently in an unblinded manner by two researchers. Disagreements were resolved through discussion and consensus.

### 4.8. Summary Measures and Analysis

This review employed a qualitative summary and narrative analysis of the retrieved results. Due to our very broad PICOS criteria, the breadth of the employed experimental models and neuroprotective interventions prevented us from performing a quantitative analysis. While we identified two distinct subsets that might lend themselves to a future quantitative meta-analysis, we decided against a statistical appraisal of only these subsets for two reasons: We judged that, since our search protocol did not specifically target these interventions, a subsequent more focused systematic database search might yield additional results, rendering a potential subset analysis within this review to be incomplete and of questionable value, and furthermore, due to our decision to include both human and animal experiments, a further exclusion of studies within the subsets would have been necessary. To preserve the internal coherence of the review, we therefore presented all results in narrative form.

## 5. Conclusions

A systematic literature search of three medical databases regarding neuroprotective strategies during hypoglycemia yielded only a small number of studies, although both human studies and animal experimental studies were included. While individual results appeared promising within the context of the respective study’s experimental design, the approaches included in this review require further research. Currently, no treatment options to reduce potential cerebral damage during hypoglycemia exist for clinical use. Efforts should be made to reduce potential sources of bias and to standardize experimental models and designs to increase the translational value of subsequent research.

## Figures and Tables

**Table 1:** General characteristics.

Reference	Model	Intervention	Total Number of Participants, *n* = *x*	Extent of Hypoglycemia	Observation Period after Intervention
[32]	Rats	4CIN vs. aCEF vs. BIC vs. DZ vs. OX, after either lactate or aCEF	38	2.5 ± 0.3 mmol/L	None
[33]	Rats	Vehicle vs. pyruvate	22	1–2 mmol/L	3 days after last recurrent hypoglycemia intervention
[34]	Rats	Hypo/EU clamp + lactate in either Ctrl or 3dRH animals	44	2.5 mmol/L	1 day
[19]	Humans	Crossover study: HYPO clamp with lactate vs. without lactate	14 (7 healthy, 7 DM)	stepwise: 4.8, 3.6, 3.0, 2.8 mmol/L	None
[18]	Humans	Crossover study: HYPO clamp with lactate vs. without lactate	7 (healthy)	stepwise: 5.0, 3.4, 2.8, 2.4 mmol/L	None
[20]	Humans	Crossover study: HYPO clamp with medium chain triglycerides vs. placebo	11	2.8 ± 0.16 mmol/L	None
[21]	Humans	Crossover study: HYPO clamp with theophylline vs. placebo	30 (15 DM with HA, 15 healthy)	stepwise: 5.0, 3.5, 2.5 mmol/L	None
[22]	Humans	MDI + SMBG vs. MDI + SMBG + RT-CGM vs. CSII + SMBG vs. CSII + SMBG + RT-CGM	18	stepwise: 5.0, 3.8, 3.4, 2.8, 2.4 mmol/L	6 months between two HYPO Clamp procedures with intervention in between
[23]	Humans	HIIT vs. rest	30	2.8 mmol/L	None
[24]	Humans	Crossover study: Human insulin vs. insulin analogue detemir	12	stepwise: 4.4, 3.7, 3.0, 2.7 mmol/L	None
[35]	Rats	Hypothermia + halothane vs. hypothermia + isoflurane vs. normothermia + halothane vs. normothermia + isoflurane	32	isoelectricity in EEG	4 hours up to 7 days
[25]	Humans	Crossover study: Glibenclamide vs. diazoxide vs. placebo	10	stepwise: 5.0, 3.4, 2.8, 2.4 mmol/L	None
[36]	Rats	Blood glucose reperfusion to ≤3 mmol/L vs. ≤6 mmol/L vs. ≤9 mmol/L vs. >9 mmol/L	30	<1 mmol/L	7 days
[37]	Rats	Cyclosporin A vs. FK506	9	isoelectricity in EEG	30 min up to 2 days
[38]	Rats	Cylosporin A (varying doses) vs. FK506	66	isoelectricity in EEG	7 days
[26]	Humans	Sleep deprivation vs. normal sleep	14	2.5 mmol/L	85 min
[39]	Rats	Citociline vs. vehicle	42	isoelectricity in EEG	7 days
[27]	Humans	HYPO clamp in patients with high RAS activity vs. patients with low RAS activity	18	2.5–2.0 mmol/L	60 min
[28]	Humans	Crossover study: Erythropoietin vs. placebo	11	2.2–2.0 mmol/L	30 min
[40]	Rats	NBQX vs. NBQX + dizocilpine vs. dizocilpine vs. CGP 40,116	44	isoelectricity in EEG	3–4 days
[44]	Mice	Vitamin C vs. vitamin E vs. vitamin C + vitamin E	64	<1 mmol/L	None
[41]	Rats	Insulin-treated DM vs. untreated DM	55	0.8–0.6 mmol/L	1 week up to 8 weeks
[29]	Humans	Crossover study: Human insulin vs. insulin analogue detemir	10	stepwise: 5.0, 4.3, 3.6, 3.0 mmol/L	None
[30]	Humans	Crossover study: HYPO clamp + oral amino acids vs. HYPO clamp + placebo vs. EU clamp + oral amino acids	20	2.6 mmol/L	None
[42]	Rats	CrHis vs. CrPic vs. dextrose	70	isoelectricity in EEG	1 day
[43]	Rats	Memantine vs. erythropoietin	36	0.8–0.6 mmol/L	7 days
[31]	Humans	Crossover study: HYPO clamp + modafinil vs. HYPO Clamp + placebo vs. EU clamp + modafinil vs. EU clamp + placebo	9	stepwise: 4.4, 3.8, 3.4, 2.8, 2.4 mmol/L	None

Abbreviations (in order of occurrence): 4CIN = lactate transport inhibitor; aCEF = artificial extracellular fluid; BIC = bicuculline methiodide; DZ = diazoxide; OX = oxamate; HYPO = hypoglycemic; EU = euglycemic; Ctrl = control; 3dRH = 3 days recurring hypoglycemia; MDI = multiple daily injections; SMBG = self-monitoring of blood glucose; RT-CGM = real time-continuous glucose measurement; CSII = continuous subcutaneous insulin infusion; HIIT = high-intensity interval training; RAS = renin-angiotensin system; NBQX = alpha-amino-3-hydroxy-5-methyl-4-isoxazolepropionic acid (AMPA)-receptor antagonist; DM = diabetes mellitus; CrhHis = chromium histidinate; CrPic = chromium29 picolinate.

**Table 2:** Outcome parameters.

Reference	Vital Parameters (Hemodynamics, Blood Pressure, Heart Rate, Temperature) Blood Analysis (Blood Gases, Metabolic Products)	Counter-Regulatory Hormones (Catechol-Amines, Glucagon, Growth Hormone, Cortisol)	Brain-Specific Parameters (CBF, EEG, etc.)	Brain Section Staining/ Histopathology)	Neuro-Proteins and Receptors, Apoptosis Markers	Cognitive Function Tests	Symptom Assessment (Autonomic and Neuroglycopenic)
[32]	-	+	-	-	GABA	-	-
[33]	GSH, Zn	-	-	+	-	-	-
[34]	+	-	CBF, EEG	-	GLUT1, GLUT2, GLUT3	-	-
[19]	+	+	-	-	-	4-CRT	+
[18]	-	+	-	-	-	4-CRT	+
[20]	+	+	-	-	-	DS, DSS, WMS	+
[21]	+	+	CBF	-	-	-	+
[22]	+	+	-	-	-	4-CRT, Str	+
[23]	+	+	-	-	-	DS, VF, PASAT	+
[24]	+	+	-	-	-	Str, VRT, VM	+
[35]	+	-	-	+	-	-	-
[25]	-	+	-	-	-	4-CRT, Str, FT	+
[36]	-	-	-	+	-	-	-
[37]	+	-	-	-	Cas3, AIF, Cyt-c,	-	-
[38]	-	-		+	MRR	-	-
[26]	-	-	-	-	-	4-CRT, DSS, NART, WQ, MT	+
[39]	+	-	EEG	+	CHAT	-	-
[27]	+	-	-	-	-	AQT, CCAP	-
[28]	+	+	ÊEG	-	-	CCAP, TM, Str	+
[40]	+	-	-	+	-	-	-
[44]	+	-	-	-	MAD, SOD, GSHPx	-	-
[41]	-	-	-	+	-	LA, SM, MWM	-
[29]	+	+	-	-	-	TM, VM, DS, Str, PASAT	+
[30]	+	+	-	-	-	TM, VF, VM, DV, DS, Str, PASAT	+
[42]	+	-	-	-	MAD, GAP43, NCAM, GLUT1, GLUT3, NF-KB, HNE, Nrf2	-	-
[43]	-	-	-	+	-	-	-
[31]	+	+	-	-	-	4-CRT, FT, Str	+

Abbreviations of outcome parameters: GABA = gamma-amino butyric acid; GSH = glutathione; Zn = Zinc; CBF = cerebral blood flow; EEG = electroencephalography; GLUT = glucose transporter; 4-CRT = 4 choice reaction time; DS = digit span test; DSS = digit symbol substitution test; WMS = Wechsler memory scale; Str = Stroop test; VF = verbal fluency; PASAT = Paced auditorial serial addition test; VRT = Vienna reaction time; VM = verbal memory test; FT = finger tapping; Cas3 = Caspase 3; AIF = apoptosis-inducing factor; Cyt-c = Cytochrome c; MRR = mitochondrial respiratory rate; NART = national adult reading test; WQ = willpower questionnaire; MT = memory tests; Chat = choline acetyltransferase; AQT = Alzheimer quick test; CCAP = California cognitive assessment package; TM = trail-making test; MAD = malondialdehyde; SOD = superoxide dismutase; GSHPx = glutathione peroxidase; LA = locomotor activity tests; SM = sensorimotor tests; MWM = Morris water maze test; DV = digit vigilance test; GAP43 = growth-associated protein 43; NCAM = neural cell adhesion molecule; NF-KB = nuclear factor kappa; HNE = 4-hydroxyl nonenal; Nrf2 = nuclear factor (erythroid-derived 2)-like 2.

**Table 3:** Specific characteristics of energy substitution interventions.

Reference	Model	Intervention	Dosage	Start of Intervention Respective to Hypoglycemia	Length of Subsequent Observation Period
[32]	Rats	Lactate transporter blockade (4CIN), GABA receptor antagonist (BIC), KATP channel blockade (diazoxide), lactate dehydrogenase inhibitor (OX)	4CIN = 15 nmol; BIC = 12.5 pmol; diazoxide = 1 nmol; OX = 50 nmol	Immediately before hypoglycemic clamp	None
[33]	Rats	Pyruvate	500 mg/kg	10 min after termination of daily recurrent hypoglycemia (5 days)	3 days
[34]	Rats	0.35M [3-^13^ C] lactate	Initial bolus of 1370 µL/kg body weight, thereafter stepwise reduction from 428 µL/min/kg to 162.8 µL/kg/min over 20 min, thereafter continuous 162.8 µL/kg/min	Immediately after reaching target glucose level	1 day
[18]	Humans	Lactate	Continuous 30 µmoL/kg/min	40 min before hypoglycemia	None
[19]	Humans	Lactate	Continuous 30 µmoL/kg/min	After reporting of first neuroglycopenic response	None
[20]	Humans	Medium-chain fatty acids	Total of 40 g (in 25-min intervals: 20 g, 10 g, and 10 g)	First ingestion 5 min before hypoglycemia	None

**Table 4:** Specific characteristics of hypoglycemia awareness interventions.

Reference	Model	Intervention	Dosage	Start of Intervention Respective to Hypoglycemia	Length of Subsequent Observation Period
[21]	Humans	Theophylline	2.8 mg/kg	Immediately before hypoglycemia	None
[22]	Humans	MDI + SMBG vs. MDI + SMBG + RT-CGM vs. CSII + SMBG vs. CSII + SMBG + RT-CGM	Application in daily routine	After the first hypoglycemic procedure	Six months application, thereafter second hypoglycemic experiment
[23]	Humans	High-intensity interval training	~15 mins on cycle ergometer: three 4-min periods at 50 W, interspersed with three 30-s all-out sprints	Before hypoglycemia	None
[29]	Humans	Human insulin vs. insulin detemir	Human insulin: Bolus of 10 mU/kg, followed by 240 min of 1 mU/kg/min and 30 min of 2 mU/kg/min; insulin detemir: Bolus of 20 mU/kg, followed by 2 mU/kg/min and 30 min of 4 mU/kg/min	Throughout the entire experiment	None
[24]	Humans	Human insulin vs. Insulin detemir	Human insulin: Bolus of 60 mU/kg, followed by continuous infusion of 2 mU/kg/min; insulin detemir: Bolus of 660 mU/kg, followed by a continuous infusion of 5 mU/kg/min	Throughout the entire experiment	None

**Table 5:** Specific characteristics of other neuroprotective strategies.

Reference	Humans/Animal Model	Intervention	Dosage	Start of Intervention Respective to Hypoglycemia	Length of Subsequent Observation Period
[35]	Rats	Hypothermia	33 °C for 30 min	Immediately before establishment of hypoglycemia	4 h up to 7 days
[25]	Humans	Glibenclamide vs. Diazoxide	Glibenclamide: 10 mg; diazoxide: 5 mg/kg	45 min before hypoglycemia	None
[36]	Rats	Differing blood glucose levels post-hypoglycemia	Infusion of 25% glucose solution until target level was reached	Immediately following the hypoglycemic procedure	7 days
[38]	Rats	CsA vs. FK 506	CsA: Either 20mg/kg or 50mg/kg; FK506: 2mg/kg	~30 min before onset of isoelectric EEG	7 days
[37]	Rats	CsA vs. FK 506	CsA: 50mg/kg; FK506: 2mg/kg	~30 min before onset of isoelectric EEG	30 min up to 2 days
[26]	Humans	Sleep deprivation	1 night of sleep deprivation	Night before the hypoglycemic clamp	85 min
[39]	Rats	Citociline	500 mg/kg	Immediately after hypoglycemia	7 days
[27]	Humans	Measurement of EPO and RAS activity	/	/	60 min
[28]	Humans	EPO	40,000 IU	6 days before hypoglycemia	30 min
[43]	Rats	EPO vs. memantine	EPO: 5000 IU/kg on three occasions; memantine: 20 mg/kg	EPO: 24 h before and after hypoglycemia (ip), Immediately after hypoglycemia (iv); memantine: Immediately after hypoglycemia	7 days
[40]	Rats	NBQX vs. NBQX + dizocilpine vs. dizocilpine vs. CGP 40116	NBQX: 30 mg/kg (ip), followed by 225 µL/kg/min for 6 h i.v.; NBQX + dizolcilpine: 10 mg/kg (ip), followed by 225 µL/kg/min for 6 h i.v. + 2 x 0.33 mg/kg; dizolcilpine: 1 mg/kg (iv); CGP40116: 10 mg/kg (ip)	All immediately after hypoglycemia, except CGP40116 (during EEG isoelectricity)	3–4 days
[44]	Mice	Vitamin C vs. vitamin E	Vitamin C: 1000 mg/kg/day; vitamin E: 100 mg/kg/day	Previous days	None
[41]	Rats	Antecedent glycemic control (with insulin)	2 U/day (target level: 100–250 mg/dL)	Three weeks before hypoglycemia	Either 1 or 8 weeks
[30]	Humans	Oral amino acids		At the beginning of hypoglycemia	None
[42]	Rats	CrHis vs. CrPic	8 µg orally per day for 7 days	7 days before hypoglycemia	1 day
[31]	Humans	Modafinil	100 mg orally	Evening before intervention	None

**Table 6:** SYRCLE’s risk of bias tool.

Reference	Sequence Generation	Baseline Characteristics	Allocation Concealment	Random Housing	Binding (Intervention)	Random Outcome Assessment	Blinding (Outcome)	Incomplete Outcome Data	Selective Outcome Reporting	Other Sources of Bias
[35]	-	+	-	-	+	+	+	+	+	+
[32]	-	+	-	?	+	+	?	+	+	+
[33]	-	+	-	?	-	+	+	+	+	+
[36]	?	+	?	-	+	?	+	+	+	-
[37]	-	+	-	?	?	?	+	+	+	?
[38]	-	+	-	?	?	+	?	+	+	?
[34]	-	+	-	?	?	-	?	+	+	-
[39]	-	+	-	?	+	-	+	+	+	+
[40]	-	+	-	?	-	+	+	+	+	+
[44]	?	+	?	N.A.	-	+	+	+	+	+
[41]	?	+	?	-	?	?	?	+	+	+
[42]	?	+	?	-	-	+	+	+	+	+
[43]	?	+	?	?	-	+	+	+	+	+

(+) indicates low risk of bias; (−) indicates high risk of bias; (N.A.) Not applicable; (?) indicates unclear risk of bias.

**Table 7:** Cochrane risk of bias tool.

Reference	Random Sequence Generation	Allocation Concealment	Blinding of Participants and Personnel	Blinding of Outcome Assessment	Incomplete Outcome Data	Selective Reporting	Other Bias
[19]	+	?	+	+	+	+	+
[18]	?	?	+	+	+	?	?
[20]	?	?	+	+	+	+	+
[21]	?	?	+	+	+	+	+
[22]	+	?	?	+	?	+	+
[23]	+	+	?	+	?	+	+
[24]	?	?	+	+	+	+	+
[25]	?	?	+	+	+	+	+
[26]	?	?	?	+	+	+	+
[27]	?	?	+	+	+	+	+
[28]	+	+	+	+	+	+	+
[29]	+	?	+	+	+	+	+
[30]	+	?	+	+	+	+	+
[31]	?	?	+	+	+	+	?

(+) indicates low risk of bias; (?) indicates unclear risk of bias.

## Appendix A

*PubMed Search Strategy*

1. hypoglycemia [MeSH terms]
2. hypoglycemic encephalopathy
3. hypogly*
4. blood sugar
5. insulin
6. 1 OR 2 OR 3 OR 4 OR 5
7. effects, neuroprotective [MeSH terms]
8. neuroprotect*
9. brain
10. cognit*
11. cerebral
12. 7 OR 8 OR 9 OR 10 OR 11
13. rct
14. trial
15. random*
16. experimental
17. 13 OR 14 OR 15 OR 16
18. 6 AND 12 AND 17

*Web of Science Search Strategy*

1. hypoglyc*mia
2. hypoglyc*mic encephalopathy
3. hypoglyc*
4. blood sugar
5. 5. insulin
6. diabet*
7. antidiabet*
8. 1 OR 2 OR 3 OR 4 OR 5 OR 6 OR 7
9. neuroprotect*
10. brain
11. cerebral
12. cognit*
13. neuro*
14. 9 OR 10 OR 11 OR 12 OR 13
15. random*
16. rct
17. trial
18. experimental
19. 15 OR 16 OR 17 OR 18
20. 8 AND 14 AND 19

*CENTRAL Search Strategy*

1. hypoglycemia [MeSH descriptor]
2. hypogly*
3. blood sugar
4. insulin
5. #1 or #2 or #3 or #4
6. neuroprotective agents [MeSH descriptor]
7. neuroprotect*
8. brain
9. cerebral
10. cognit*
11. #6 or #7 or #8 or #9 or #10
12. #5 and #11
